# Iron Deficiency as an Underrecognized Cause of Dyspareunia and Vulvar Bleeding

**DOI:** 10.7759/cureus.94659

**Published:** 2025-10-15

**Authors:** Mehtab Grewal, Maryam Rehan, Deepika Davalath, Parth Patel, Rumana Tokaria

**Affiliations:** 1 Hematology and Medical Oncology, AtlantiCare Regional Medical Center, Atlantic City, USA; 2 Internal Medicine, AtlantiCare Regional Medical Center, Atlantic City, USA

**Keywords:** anemia, dyspareunia, iron deficiency anemia (ida), vaginal atrophy, womens health

## Abstract

Iron deficiency is a common cause of anemia and is associated with a range of systemic effects. However, its direct impact on vulvovaginal tissue remains poorly described in the literature. We report the case of a 39-year-old woman with a history of gastric bypass surgery who presented with a 2-year history of severe dyspareunia and vulvar and vaginal mucosal tearing, including bleeding with minimal trauma, such as wiping after urination. The patient was seen by Gynecology for these symptoms, and a vulvar biopsy was suggested. Simultaneously, the patient was being treated for iron deficiency anemia by her primary care provider. Following intravenous iron replacement, the patient experienced significant resolution of dyspareunia and vulvar bleeding. This case suggests a potential association between iron deficiency and vulvovaginal mucosal atrophy or fragility. While iron deficiency is known to affect other mucosal tissues, its role in gynecologic symptoms, such as dyspareunia, has not been widely explored.

## Introduction

Iron deficiency is highly prevalent worldwide. In 2021, the global age-standardized prevalence of dietary iron deficiency was 16,434.4 per 100,000 population (16.7%), with a disproportionately higher burden among female individuals [1]. Iron deficiency can progress to iron deficiency anemia (IDA), which may present with symptoms such as poor appetite, fatigue, exercise intolerance, dizziness, tachycardia, shortness of breath, and diaphoresis. Common physical examination findings include dry skin, atrophic glossitis, cheilosis, nail changes, and alopecia. Iron deficiency has also been associated with pica, restless leg syndrome, mood disturbances, and hearing loss [2]. An iron-deficient state has also been associated with several adverse health consequences when it comes to women's health, especially its significant impact on sexual health in women [3].

## Case presentation

We present the case of a 39-year-old woman with a medical history significant for gastric bypass surgery, gastroesophageal reflux disease (GERD), dyslipidemia, renal stones, and recurrent urinary tract infections. Since the birth of her second child, she has suffered from severe dyspareunia, along with vaginal and vulvar mucosal tearing. She reported bleeding even from wiping after urination. These symptoms persisted for two years, significantly affecting her intimate relationship.

In June 2024, the patient consulted a gynecologist due to ongoing symptoms. Examination revealed an atrophic vulva. Hormonal evaluation, including estradiol, follicle-stimulating hormone (FSH), luteinizing hormone (LH), thyroid-stimulating hormone (TSH), and free thyroxine (T4) levels, was within normal limits. The patient was advised to apply Aquaphor or coconut oil to the vulvar area twice daily for symptomatic relief, and a vulvar biopsy was recommended.

Around the same period, laboratory results from April 2024 showed a hemoglobin level of 10 g/dL, mean corpuscular volume (MCV) of 74 fL, serum iron of 19 µg/dL, total iron-binding capacity (TIBC) of 421 µg/dL, iron saturation of 5%, and a ferritin level of 4 ng/mL--findings consistent with iron deficiency anemia. She was initially started on oral ferrous sulfate 325 mg delayed-release tablets (65 mg elemental iron) once daily with meals, taken alongside vitamin C or orange juice to enhance absorption. However, repeat labs in June revealed persistently low iron indices. As a result, in July, she was transitioned to intravenous iron therapy with ferric carboxymaltose (Injectafer) 750 mg IV, administered in 2 doses 1 week apart, for a total cumulative dose of 1,500 mg.

Following treatment with IV iron therapy, the patient experienced significant improvement in both dyspareunia and vulvar bleeding, alleviating her symptoms and enhancing intimacy with her partner (Table 1).

**Table 1:** Lab work comparison pre and post IV iron transfusion therapy RBC: Red Blood Cells; MCV: Mean Corpuscular Volume; MCH: Mean Corpuscular Hemoglobin; MCHC: Mean Corpuscular Hemoglobin Concentration; RDW: Red Cell Distribution Width; TIBC: Total Iron-Binding Capacity; UIBC: Unsaturated Iron-Binding Capacity

Labs	6/15/2024 (Pre-IV transfusion therapy)	9/25/2024 (Post-IV iron transfusion therapy)	Reference range
RBC	4.47	4.92	3.77x10E6/uL-5.28x10E6/uL
Hemoglobin	9.2	13.5	11.1g/dL-15.9g/dL
Hematocrit	32.4	42.2	34%-46%
MCV	73	86	79fL-97fL
MCH	20.6	27.4	26.6pg-33pg
MCHC	28.4	32	31.5g/dL-35.7g/dL
RDW	14.8	19.1	11.7%-15.4%
Serum Iron	16	78	27ug/dL-159ug/dL
TIBC	412	291	250ug/dL-450ug/dL
Iron saturation	4	27	15%-55%
Ferritin	3	76	15ng/mL-150ng/mL
UIBC	396	213	131ug/dL-425ug/dL

## Discussion

Iron deficiency is well-documented to impact sexual function in women, often attributed to associated symptoms such as fatigue, irritability, and anxiety [4,5]. However, the direct effects of iron deficiency on vaginal and vulvar tissue have not been thoroughly investigated. In this case, the patient experienced complete resolution of dyspareunia and vulvar/vaginal bleeding following iron repletion therapy, suggesting a potential correlation between iron deficiency and the mucosal integrity of the vaginal and vulvar lining.

A strength of this case is the multidisciplinary approach involving both gynecologic and primary care evaluations, alongside careful documentation of clinical response to a clearly defined intervention--intravenous iron therapy. Objective hematologic improvement was tracked through serial laboratory tests, which correlated well with the patient’s subjective symptom resolution, strengthening the temporal association between iron repletion and mucosal healing. Furthermore, normal hormonal levels reduced the likelihood of estrogen deficiency contributing to the symptoms.

Iron deficiency anemia is known to cause mucosal changes in other tissues, including thinning of the oral mucosa, atrophic glossitis, and cheilosis [6]. It is plausible that similar mechanisms affect vaginal tissue, leading to atrophy, mucosal fragility, and symptoms such as tearing and dyspareunia. This case may be among the first to document resolution of vulvovaginal mucosal symptoms following iron therapy, highlighting a potentially underrecognized clinical manifestation.

However, the observational and retrospective nature of this report limits causal inference. Additionally, no histopathological confirmation or validated sexual function assessments were performed, which would strengthen the clinical evidence.

This case suggests a testable hypothesis: iron deficiency independently contributes to vulvovaginal mucosal atrophy or fragility, and iron repletion can improve mucosal integrity and sexual function in affected patients. Future prospective studies using histologic evaluation and validated sexual function questionnaires would be valuable to investigate this relationship further.

Given the affordability and simplicity of iron studies, screening for iron deficiency should be considered in the assessment of women presenting with sexual dysfunction and unexplained vulvovaginal symptoms [7].

## Conclusions

This case highlights a potentially under-recognized link between iron deficiency anemia and vulvovaginal mucosal fragility, which may manifest as dyspareunia and vulvar/vaginal bleeding. It supports the need for broader awareness and further research into the gynecologic manifestations of iron deficiency. Considering iron deficiency as a potential differential diagnosis in patients presenting with symptoms such as dyspareunia and vulvar bleeding may help clinicians alleviate patient suffering and reduce the need for unnecessary or extensive testing.
